# Clinical Profile of Patients with Interstitial Lung Disease with Underlying Autoimmune Rheumatic Disease Presenting to a Tertiary Care Setting: A Descriptive Cross-sectional Study

**DOI:** 10.31729/jnma.v63i292.9268

**Published:** 2025-12-31

**Authors:** Ujjwol Prasad Risal, Rakshya Pandey, Raju Prasad Pangeni

**Affiliations:** 1 Department of Internal Medicine, Hospital for Advanced Medicine and Surgery, Kathmandu, Nepal; 2 Department of Pulmonology and Sleep Medicine, Hospital for Advanced Medicine and Surgery, Kathmandu, Nepal

**Keywords:** *autoimmune rheumatic disease*, *clinical profile*, *interstitial lung disease*

## Abstract

**Introduction:**

Interstitial lung diseases are a diverse group of disorders that display considerable overlap clinically, physiologically, and radiologically. A significant subset of these patients has autoimmune rheumatic diseases. This study aimed to characterize the clinical profile of patients with autoimmune rheumatic disease-related interstitial lung disease, focusing on demographic features, radiographic patterns, and treatment practices.

**Methods:**

A descriptive cross-sectional study was conducted at the Hospital for Advanced Medicine and Surgery in Kathmandu, Nepal, from May 2022 to May 2023. Patients with interstitial lung disease confirmed by high-resolution computed tomography and a rheumatologist-verified diagnosis of autoimmune rheumatic disease were included. Demographic, clinical, laboratory, imaging, and pulmonary function data were collected and analysed.

**Results:**

A total of 36 patients were included, comprising 28 (77.80%) females and 8 (22.20%) males, with a mean age of 58.92 ± 15.89 years. Rheumatoid arthritis was present in 18 (50%) cases, while undifferentiated systemic rheumatic disease was seen in 7 (19.44 %). Extrapulmonary manifestations were the initial presentation in 24 (66.7%) of cases. Usual interstitial pneumonia was identified in 22 (61.1%) patients. The mean forced vital capacity was 65.90 ± 25.38%, and the mean diffusion capacity of the lungs for carbon monoxide was 47.22 ± 23.21%. Methotrexate and hydroxychloroquine were each used by 6 (16.70%) patients, while corticosteroids were used by 11 (30.60%) patients.

**Conclusions:**

Interstitial lung disease is a significant complication of autoimmune rheumatic diseases. Early identification through regular clinical assessment and imaging is essential to prevent disease progression and improve outcomes.

## INTRODUCTION

Interstitial lung diseases (ILDs) encompass more than 200 diseases with considerable clinical, physiological, and radiological overlap. Broadly, they can be subdivided into those with an identifiable cause and those without. an identifiable cause.^1^ Autoimmune rheumatic diseases (AIRD) are heterogeneous disorders that mainly affect joints and muscles and include rheumatoid arthritis (RA), systemic lupus erythematosus (SLE), primary Sjogren’s syndrome (PSS), systemic sclerosis, idiopathic inflammatory myopathies (IIM), and the systemic vasculitides.^2^ ILD is a common manifestation of most of these disorders. Approximately 15% of patients presenting with ILD have an underlying AIRD.^3^ ILD may precede, accompany, or represent the sole manifestation of an underlying AIRD.^4^ The prevalence of ILD varies across AIRDs with higher occurrence in systemic sclerosis and IIMs.^5^ AIRD-related ILDs usually have a better prognosis and respond well to immunosuppressives.^6^ The primary goal of this research is to describe the clinical profile of patients with AIRD-related ILD.

## METHODS

This hospital-based descriptive cross-sectional study was conducted at Hospital for Advanced Medicine and Surgery (HAMS), a tertiary care hospital in Kathmandu, Nepal. Ethical approval was obtained from the Ethical Review Board (Reference number:3025). Data were collected from all patients with ILD and AIRD who presented to the rheumatology or pulmonology outpatient clinics or who were admitted to the wards in these departments from May 12, 2022, to May 11, 2023. Since ILD related to AIRDs is rare, all patients who presented within the one-year study period of one year were included. All patients aged 18 and above with a confirmed diagnosis of ILD based on high-resolution computed tomography (HRCT) and an underlying AIRD were included in the study. Patients with ILD but without associated AIRD, and those who declined to provide consent, were excluded.

ILD was defined based on the HRCT findings evaluated by experienced pulmonologists and radiologists at HAMS. AIRD was defined based on the diagnosis made by the rheumatologist working at HAMS. Demographics, duration of illness, current medications, clinical examination findings, laboratory investigations, HRCT thorax findings, echocardiography findings (for pulmonary hypertension), type of AIRD, pulmonary function test (PFT) results, including diffusion capacity of the lungs for carbon monoxide (DLCO), were recorded. Data were entered into Microsoft Excel 2016, and analysis was conducted using IBM Statistics SPSS 23.0. This work has been reported in line with the STROCSS criteria.^7^

## RESULTS

A total of 36 patients were included in the study. There were 28 (77.80%) females and 8 (22.20%) males with a mean age of 58.92± 15.89 years and a mean body mass index (BMI) of23.85 kg/m^2^ (Table 1). Regarding smoking status, 24 (66.70%) were non-smokers, 10 (27.80%) were reformed smokers, and 1(2.8%) was an active smoker. The mean duration of illness was 64.19 ±82.24 months (Table 1).

**Table 1:** Clinical Characteristics of patients with ILD with AIRD (n=36).

Parameters	n(%)
**Sex**	
Male	8(22.20)
Female	28(77.80)
**Predominant symptom at presentation**	
Pulmonary	10(27.80)
Extrapulmonary	24(66.70)
Both	2(5.55)
**Smoking status**	
Smoker	1(2.77)
Non-smoker	10(27.80)
Reformed smoker	24(66.70)
Raynaud’s phenomenon	5(13.88)
**Symptomatic**	
Cough	33(91.67)
Dyspnea	33(91.67)
Clubbing	2(5.55)
Velcro crackles	34(94.44)
Pulmonary hypertension	5(13.88)
Abnormal Chest X-ray	36(100)

**Table 2:** Frequency of Autoantibodies (n=36).

Antibody	n(%)
Rheumatoid factor	17(47.22)
Anti-CCP	16(44.44)
Antinuclear antibody	12(33.33)
U1RNP	3(8.33)
Anti Scl 70	2(5.55)
SSA	7(19.44)
SSB	2(5.55)
Anticentromere antibody	1(2.77)
Anti MDA-5 antibody	1(2.77)

U1RNP = U1-Ribonucleoprotein Antibody; Anti-Scl-70 = Anti-Topoisomerase I Antibody; SSA = Anti-Ro Antibody; SSB = Anti-La Antibody; Anticentromere Antibody = Anticentromere Antibody; Anti-MDA-5 = Anti-Melanoma differentiation-associated gene 5 Antibody; Anti-CCP = Anti-Cyclic citrullinated peptide Antibody.

**Table 3:** Predominant Pattern on HRCT Chest (n=36).

HRCT Pattern	n(%)
UIP	17(47.22)
Probable UIP	4(11.11)
Indeterminate UIP	1(2.77)
NSIP	9(25)
Bronchiolitis	1(2.77)
Organizing pneumonia	1(2.77)
Unclassified	3(8.3)

HRCT = High-Resolution Computed Tomography; UIP = Usual Interstitial Pneumonia; NSIP = Nonspecific Interstitial Pneumonia;

**Table 4:** Pulmonary function tests and DLCO of AIRD-related ILD Patients (n=36).

Parameters	n(%)
**Spirometry pattern**	
Normal	10(27.78)
Restrictive	17(47.22)
Obstructive	3(8.33)
Unable to perform PFT	6(16.67)
Missing	1(2.77)
Mean FVC, % predicted	65.90 ± 25.38
Mean DLCO, % predicted	47.22 ± 23.21
FCV= Forced Vital Capacity; DLCO= Diffusing Capacity of the Lungs for Carbon Monoxide.	

Rheumatoid Arthritis accounted for 18 (50%) cases, followed by undifferentiated systemic rheumatic disease (USRD) in 7 (19.40%) patients. Systemic sclerosis was 4 (11.11%), Mixed Connective T issue Disease (MCTD) 2 (5.56%), Primary Sj ogren’s Syndrome (PSS) 3 (18.33%) and Palindromic Rheumatism in 1 (2.77%). Hypertension and hypothyroidism were present in 11 (30.55%) patients each. The predominant symptoms at presentation were extrapulmonary in 24 (66.70%) patients, pulmonary in 10 (27.80%), and both in 2 (5.60%). Rheumatoid factor (RF) was positive in 17 (47.20%) patients, anti-cyclic citrullinated peptide (anti-CCP) antibody in 16 (44.40%), and antinuclear antibody (ANA) in 13 (36.10%) (Table 2).

Usual interstitial pneumonia (UIP) was seen in 22 (61.10%) patients with (non-specific interstitial pneumonia (NSIP) in 9 (25%) (Table 3). Methotrexate and hydroxychloroquine were each used in 6 (16.7%) patients, leflunomide in 5 (13.8%), and corticosteroids in 11 (30.6%) patients, with a mean prednisolone dose of 14.1 mg. Pulmonary function testing showed a restrictive pattern in 17 (47.2%) patients, with a mean forced vital capacity (FVC) of 65.9 ± 25.4% and DLCO of 47.2 ± 23.2% (Table 4).

## DISCUSSION

There was a predominance of females in our study, which is due to the fact that AIRDs tend to affect females more than males. This finding is similar to the study done by Agarwal et.al., where 78% of the 100 patients were female.^8^ The mean age of our sample (58.92 years) was higher than that of the study by Agarwal et.al., which was 45.69 years.^8^ This could be explained by the fact that the mean duration of illness in our study was around 64 months, suggesting that the onset of the disease could be similar to other studies.

Regarding smoking status, the majority of patients were nonsmokers, with a small percentage being former smokers. This finding is encouraging and highlights that most patients are aware of the harmful effects of smoking on their disease. RA was the most common AIRD associated with ILD, comprising 50% of the samples. Other similar studies have also shown that RA-ILD is the most common form of AIRD-related ILD.^8-10^ Even though the frequency of ILD among patients with RA is low, RA is the most common cause of chronic inflammatory joint disease and therefore commonly seen.^11^ USRD was the second most common form of AIRD associated with ILD in our study. It is possible that the current diagnosis of USRD may change, as connective tissue diseases tend to evolve into a distinct disease over time.

Our study also showed that the extra-pulmonary symptoms were the presenting symptoms in the majority (66.7%), followed by pulmonary symptoms in about 27% and both in about 10%. This finding is consistent with various studies suggesting that ILDs are diagnosed after a diagnosis of AIRD most of the time, but can occur concomitantly or years before the diagnosis of an AIRD.^12^ Around 91% of patients had symptoms of cough and exertional dyspnoea, and the rest were only detected incidentally, which is consistent with various other studies.^13,14^

Raynaud’s phenomenon (RP) was seen in only 13.88% of patients, likely because most of the patients had RA ILD, and RP is not very common in RA. Clubbing was only seen in 5.55 %, which is much lower than in other similar studies.^8,15^ This difference could be because clubbing is a late finding of ILD, suggesting advanced fibrosis, and most of our patients were in the early stages of the disease. Velcro crackles were seen in almost 95% of our patients, which is higher than in another study done in similar settings.^8^ Pulmonary hypertension (pulmonary artery systolic pressure ≥ 40 mm Hg) was seen in 13.88% patients, consistent with a similar study done in Portugal but much less than another study done in India.^8,16^ As most of the patients had RA and were in the early stage of ILD, there was a decreased incidence of pulmonary hypertension in our study. All the patients in our study had abnormal chest X-rays, which contrasts with another similar study that showed X-ray abnormalities in only 53% of the patients.^8^

Based on HRCT findings, UIP was the most common pattern, seen in 47.22 % patients, followed by NSIP in 25%. This finding differs from other similar studies that have shown NSIP as the most common pattern, followed by UIP.^8,15,16^ This difference may be because most of our patients had RA, and UIP pattern is commonly encountered in RA ILD.^17^ Among 18 patients with RA, 14 (77.77%) had UIP, 2 (11.11%) had NSIP, and 1 (5.55%) each had organising pneumonia and bronchiolitis. Among 4 patients of systemic sclerosis, 3 (75%) had UIP and 1 (25%) had NSIP. Among the 7 patients of USRD, 3 (42.85%) had NSIP, 1 (14.2%) had UIP, and 3 (42.85%) had an unclassified pattern on HRCT. Of the 2 patients with MCTD, 1 each had UIP and NSIP patterns. Of the 3 patients with PSS, 2 had UIP and 1 had NSIP.

Regarding immunosuppressive drugs, most of the patients were on methotrexate and hydroxychloroquine, followed by leflunomide. After the diagnosis of ILD, methotrexate was stopped in a few patients who had severe fibrosis due to the fear of methotrexate induced lung injury. Three patients were on tofacitinib, and one was on rituximab. Thirty percent of patients were on steroids, with a mean prednisolone dose of 14.06 mg per day. Hypertension and hypothyroidism were the most common comorbidities seen in our patients, followed by diabetes mellitus, which is consistent with other studies.^15^ One patient with small vessel vasculitis had autoimmune hemolytic anemia. One patient with RA had adrenal insufficiency, and one patient with PSS had pemphigus foliaceous. Nine (25%) patients did not have any comorbidities.

Spirometry was suggestive of a restrictive defect with a moderate reduction in DLCO in the majority of patients, which is consistent with other studies.^8,15,16^ Six patients were unable to perform spirometry, and 12 patients were unable to perform the DLCO test. There was missing data for spirometry and DLCO for one patient.

Our study was the first of its kind done in Nepal with the largest number of AIRD-ILD patients in a single centre over one year, given the rarity of AIRD-associated ILD. However, our study had some limitations. It was a single-centre study with a relatively small sample size and short duration. Arterial blood gas analysis and 6-minute walk test data were not included. No biopsy of the lungs was done to confirm the ILD pattern, which is often unwarranted in CTD-ILDs.

## CONCLUSIONS

The most predominant Interstitial lung diseases in Autoimmune rheumatic diseases was Usual Interstitial Pneumonia(UIP). One third patient had pulmonary symptom on presentation.

## Data Availability

The data are available from the corresponding author upon reasonable request.
